# The effect of Dynamic tape’s directional support on shoulder fatigue and pitching performance in amateur baseball players: a randomized crossover trial

**DOI:** 10.1186/s13102-024-00965-8

**Published:** 2024-08-23

**Authors:** Haw-Ming Huang, Chih-Yang Hsu, Ting-Hsuan Hsu, I-Fan Hsieh, Po-Chieh Yang, Yu-Hsuan Cheng

**Affiliations:** 1 School of Dentistry, College of Oral Medicine, Taipei Medical University, Taipei, Taiwan; 2 Department of Physical Medicine and Rehabilitation, Wan-Fang Hospital, Taipei Medical University, No.111, Sec. 3, Xinglong Rd., Wenshan Dist., Taipei City, 116 Taiwan

**Keywords:** Tape, Shoulder, Strength

## Abstract

**Background:**

To evaluate whether the application of Dynamic tape to the pitching shoulder could result in reduced shoulder fatigue, reduced delayed onset muscle soreness, or improved performance.

**Methods:**

This is a randomized crossover study, in which participants and investigators were blinded, included 20 amateur adult baseball players without shoulder pain. Sham taping and Dynamic taping were randomized, using an internal rotation support taping method in both groups. Bilateral shoulder strength and range of motion were measured with a handheld dynamometer and clinical goniometer before and after each test. The percentage of strength decrease, range of motion, pitch velocity, spin rate, and shoulder pain were recorded. The post-pitching decrease in strength and percentage of strength decrease were calculated by paired t-test and the pitching speed and spin rates in the innings for both the sham and Dynamic taping groups were analyzed using two-way ANOVA.

**Results:**

Compared with the sham group, the Dynamic tape group showed a significant loss in the percentage of strength decrease in internal rotation compared to the sham group (-1.4% vs. 7.0%, *p* = 0.03). However, no significant differences were observed in other strength declines, shoulder range of motion, pain, pitching velocity, or spin rate.

**Conclusions:**

Dynamic tape reduced direction-specific shoulder fatigue but did not significantly enhance pitching performance or prevent delayed onset muscle soreness.

**Trial registration:**

ClinicalTrials: N201912094.

## Introduction

Shoulder injury is common in baseball players. In Major League Baseball, 17% of total injuries are shoulder injuries, with pitchers being the most prone to such injuries, accounting for 78% of total shoulder injuries [1]. Shoulder fatigue is a common phenomenon among pitchers and has negative effects on pitching biomechanics, increasing the risk of shoulder injury [2–4]. Maximal muscle strength decline is one of the symptoms of fatigue [5, 6]. In addition to kinematic changes in the trunk and lower extremities, decreased maximal shoulder external rotation range of motion, decreased shoulder internal rotation, flexion and abduction force, and decreased shoulder horizontal abduction torque have been reported [2, 3, 7]. These biomechanical changes have been associated with decreased ball velocity, decreased shoulder external rotation, increased arm pain, and potentially increased risk of injuries [8, 9]. Thus, preventing shoulder fatigue is an important issue to decreased pitcher’s injury.

Many strategies have been proposed in an effort to decrease shoulder fatigue and injuries, including but not limited to strength and motor training programs, form adjustments, pitch type and pitch count control, rest days, player and coach education, and taping [10–14]. Among various taping methods, Kinesio taping has been vigorously investigated [15, 16]. However, studies have not consistently supported their effectiveness in terms of fatigue reduction, biomechanical improvements, pain or injury prevention [11–13].

Dynamic Tape (DT) was designed by physiotherapist Ryan Kendrick in 2010. It is a viscoelastic tape composed of nylon and Lycra, providing the ability to stretch in four directions. This allows the tape to conform to the body and facilitate complex movements, reducing tension points that could cause blisters. Unlike Kinesio tape, which primarily acts through neurophysiological mechanisms, Dynamic Tape functions mechanically by decelerating movement, absorbing load, and assisting motion. It can stretch up to 200% without a rigid endpoint, compared to Kinesio Tape’s 140% stretch limit [17]. Studies have shown that Dynamic Tape reduces hip adduction moment, internal rotation, and pelvic obliquity displacement during walking, providing mechanical benefits and significantly reducing pain in women with greater trochanteric pain syndrome [18]. Compared to low-dye taping, Dynamic Tape also significantly reduces pain intensity from plantar fasciitis, despite not changing ankle range of motion or foot posture index [19]. Additionally, studies show that Dynamic Tape enhances muscle endurance and controls muscle fatigue in patients with low back pain by converting stored elastic potential energy into kinetic energy, thereby decreasing workload and improving biomechanical efficiency [20].

To date, no studies have evaluated the effectiveness of Dynamic taping on shoulder fatigue, range of motion and pitching performance which is including pitching speed and spin rate. Given its more pronounced mechanical properties, we hypothesize that Dynamic taping could reduce shoulder fatigue in pitchers. We therefore conducted the present study to evaluate the effects of Dynamic taping on shoulder strength, shoulder range of motion and pitching performance.

## Methods

### Experimental approach to the problem

Based on a previous study by Mullaney and considering a type 1 error of 0.05, power of 80%, 2-sided test, at least 18 participants were required [7]. We enrolled 20 participants for the current investigation. Eligible participants were individuals who were experienced in baseball, aged more than 20 years, and capable of throwing 100 pitches per game. The exclusion criteria included participants with preexisting dominant shoulder pain before pitching, those diagnosed with rotator cuff tendinopathies via sonography, or those unable to throw 100 pitches.

### Subjects

Participants were recruited from an amateur college male baseball team at Taipei Medical University between October 2020 and February 2022. All participants provided signed informed consent forms, and the protocol received approval from the Taipei Medical University Joint Institutional Review Board. All research procedures were conducted in accordance with relevant guidelines and regulations. The study was registered on Clinicaltrials.gov (NCT04504929, 07/08/2020). This study was supported by a grant from Taipei Medical University – Wan Fang Hospital (Grant No. 109TMU-WFH-20), and there were no competing interests.

### Procedures

A physician evaluated participants for inclusion, arranged testing dates, and was responsible for tape application. The dominant shoulder of each participant was assessed using sonography (minisono L3-12 linear transducer, Alpinion Medical Systems, Korea) by the same physician, who held Registered in Musculoskeletal® (RMSK®) sonography certification, to exclude rotator cuff tendinopathy before the commencement of the first test session [21]. All participants underwent two separate tests, each consisting of 5 innings and 20 pitch counts per inning. The interval between the two tests exceeded one week to ensure complete resolution of postpitching soreness and pain. A 10-min warm-up preceded each game. Due to varying biomechanics and unfamiliarity with off-speed pitches, we instructed all participants to throw only 4-seam fastballs at maximal effort for each pitch [9]. Participants rested for 15 min between innings and less than 10 s for setting, mirroring typical game conditions. This ensured realistic assessment of Dynamic Tape’s effects on shoulder fatigue and pitching performance. Before the second test, any postpitching shoulder pain following the first test must have completely subsided.

**Fig. 1:**
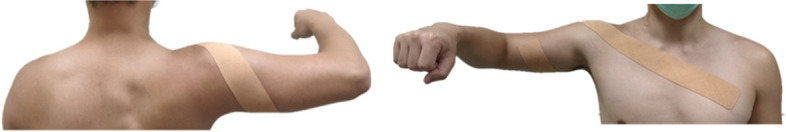
Tape-to-internal rotation support

Participants received either sham taping (elastic adhesive tape, 3 M, USA) in one game and Dynamic taping (Dynamic Tape Global®) in the other, following a crossover design, with the order determined by an investigator using computer randomization. The internal rotation support taping method was applied in both the sham and Dynamic taping groups. The dominant upper limb was initially positioned at 90 degrees shoulder abduction in the sagittal plane with the elbow flexed at 90 degrees. Participants were then instructed to perform maximal internal rotation without scapula elevation. The physician applied tape to the lateral part of the arm proximal to the elbow. Subsequently, the tape was applied superiorly at a 45-degree angle to create a spiral effect from a posterior direction over the posterior glenohumeral joint, continuing across the chest [17] (Fig. 1). The tape length was recorded at maximal internal rotation and maximal external rotation. Following the testing, we recorded the taping force. A new piece of tape was cut according to the measured length at maximal internal rotation. The tape was then stretched to the maximal external rotation length, and the generated force was measured using a digital algometer (Force Ten FDX Force Gage, Wagner Instruments, USA) to determine the internal force exerted by the tape [22, 23].

During the tests, the velocity and spin rate were recorded for every pitch. The velocity was measured using a Bushnell velocity speed gun capable of tracking ball speeds from 10 feet to 110 miles per hour (MPH) with ±1.0 MPH accuracy. The spin rate was measured using a scientific baseball training system (STRIKE Smart Baseball, Jingletek, Taiwan), which measures the spin rate of a baseball from 100 revolutions per minute (RPM) to 4000 RPM with ±85 RPM accuracy. After pitching, participants recorded the severity of their shoulder soreness daily for one week using the visual analog scale to monitor delayed onset muscle soreness [24]. At the end of the entire testing process, the participants were asked to guess which taping session utilized Dynamic tape.

Bilateral shoulder range of motion (ROM) and strength were assessed both before and immediately after the game, with participants seated and their spines in a neutral position. Tape was not applied during these assessments. ROM was measured using a clinical goniometer, while strength was evaluated using a handheld dynamometer (JAMAR Plus, Patterson Medical, Canada) with a sensitivity of 0.01 kg and calibrated according to the manufacturer’s recommendations. A handheld dynamometer, validated [25] and demonstrating good test-retest reliability by our examiner (Pearson correlation coefficient=0.96 for external rotation and 0.95 for internal rotation), was used. Shoulder fatigue was defined as a decrease in strength of maximal voluntary force [6, 7].

During the ROM tests, the participants were instructed to perform maximal active movements in shoulder flexion, extension, abduction, internal rotation, and external rotation. Shoulder flexion, extension, and abduction ROM were measured with the elbow in full extension, while internal rotation and external rotation ROM were measured with the shoulder abducted to 90 degrees in the coronal/frontal plane and the elbow flexed to 90 degrees.

The order of strength measurements was as follows: shoulder flexion, extension, abduction, internal rotation, and external rotation. Shoulder flexion and extension strength were both assessed with the shoulder at 0 degrees of flexion and the elbow in full extension. Abduction strength was assessed with the shoulder abducted to 30 degrees. The internal rotation and external rotation strength were tested with the elbow flexed to 90 degrees and the shoulder abducted to 90 degrees. After pitching, participants were asked for recording their dominant shoulder pain by numerical rating scale every day for one week.

An investigator, blinded to group assignment, was tasked with measuring shoulder ROM, shoulder strength, pitching speed, spin rate, and tape-generated force.

### Statistical analyses

Pre-pitching and post-pitching shoulder ROM, strength, and strength decreases in both the dominant and non-dominant shoulders were compared between the dynamic and sham tape groups using independent t-tests. Within each shoulder (dominant or non-dominant), comparisons of pre-pitching and post-pitching ROM and shoulder strength were conducted using paired t-tests.

Pitching speed and spin rate were averaged for each inning. The differences in pitching speed and spin rates between the sham and dynamic taping groups were analyzed using two-way ANOVA. A significance level of *p* < 0.05 was used.

Post-pitching pain from days 2 through 7 was compared to pain on day 1 using paired t-tests.

At the conclusion of the test, tape force was compared between the dynamic and sham tape groups using independent t-tests. Participants’ ability to correctly identify the tape used was assessed, and the percentage of correct identifications was calculated. The magnitude of the differences between Dynamic tape and sham tape for outcome variables was determined using Cohen’s d (effect size [ES]) [26]. When the ES was <0.2, 0.2~0.5, 0.5~0.8, or >0.8, the differences were considered trivial, small, moderate, or large, respectively [27].

## Results

**Fig. 2:**
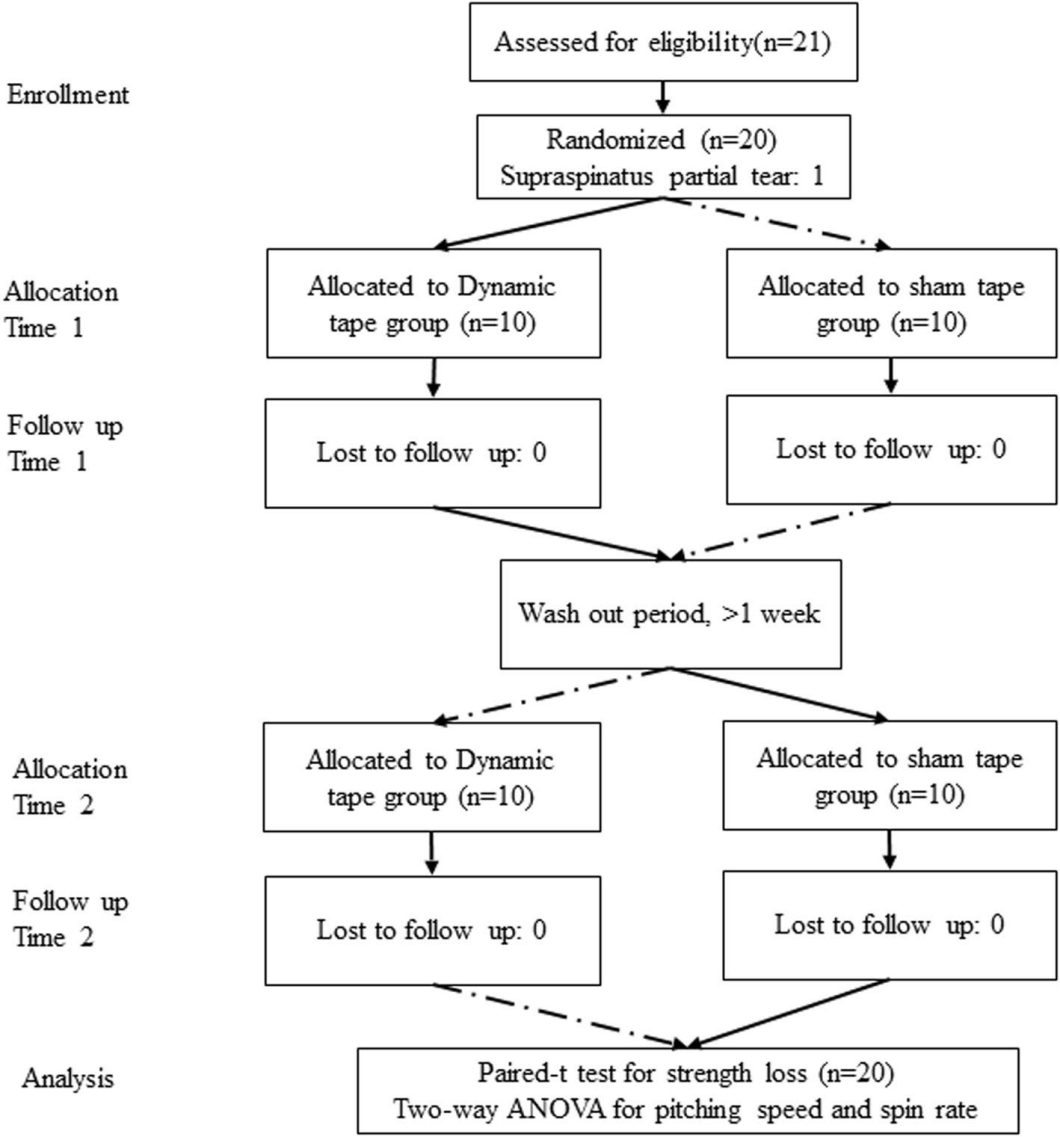
Flow diagram showing the flow of participants through each stage of the randomized crossover trial

Twenty-one participants were enrolled in this study, but one was excluded due to a sonographic supraspinatus tendon partial tear (Fig. 2). The mean age of the participants was 22.2 ± 1.6 years, with a mean body weight of 70.1 ± 11.5 kg and a mean height of 172.3 ± 4.1 cm. During baseline testing, there were no significant differences in dominant shoulder or nondominant strength or shoulder range of motion between the dynamic and sham tape groups (Tables 1 and 2).

**Table 1:** Baseline strength measurement (Pregame)

	Dynamic tape		Sham tape		DT	ST	DS	NDS
	Dominant, kg	Nondominant, kg	Dominant, kg	Nondominant, kg	DS-NDS, *p* value	DS-NDS, *p* value	DT vs ST, *p* value	DT vs ST, *p* value
Flexion	13.3 ± 3.1	12.8 ± 3.8	13.3 ± 3.3	13.2 ± 3.0	0.14	0.78	0.94	0.37
Extension	11.3 ± 3.8	10.7 ± 3.8	11.1 ± 3.3	11.0 ± 3.0	0.08	0.65	0.64	0.66
Abduction	13.0 ± 4.0	12.5 ± 3.8	12.8 ± 3.5	12.9 ± 3.6	0.05	0.63	0.69	0.32
IR	12.1 ± 3.6	11.7 ± 3.0	12.6 ± 3.4	11.6 ± 3.3	0.17	0.48	0.49	0.28
ER	11.7 ± 4.0	11.6 ± 3.0	11.2 ± 3.3	11.7 ± 3.2	0.87	0.09	0.32	0.80

Mean ± standard deviation

*Abbreviations*: *DS* dominant shoulder, *DT* Dynamic tape, *ER* external rotation, *IR* internal rotation, *NDS* nondominant shoulder, *ST* sham tape

**Table 2:** Baseline range of motion measurement (Pregame)

	Dynamic tape		Sham tape		DT	ST	DS	NDS
	Dominant	Nondominant	Dominant	Nondominant	DS vs NDS, *p* value	DS vs NDS, *p* value	DT vs ST, *p* value	DT vs ST, *p* value
Flexion	170.6 ± 5.7	172.8 ± 5.2	170.2 ± 5.5	171.3 ± 7.0	0.73	0.59	0.58	0.74
Extension	55.0 ± 10.7	54.5 ± 6.9	55.2 ± 8.8	55.2 ± 8.9	0.22	0.99	0.47	0.53
Abduction	175.6 ± 3.5	174.2 ± 5.2	172.2 ± 11.4	173.3 ± 9.2	0.68	0.76	0.64	0.15
IR	63.3 ± 14.6	67.2 ± 16.5	60.9 ± 17.2	69.2 ± 28.0	0.77	0.28	0.25	0.56
ER	116.9 ± 12.6	110.1 ± 13.6	113.9 ± 15.2	108.6 ± 10.8	0.63	0.22	0.48	0.20

Mean ± standard deviation

*Abbreviations*: *DS* dominant shoulder, *DT* Dynamic tape, *ER* external rotation, *IR* internal rotation, *NDS* nondominant shoulder, *ST* sham tape

Posttest internal rotation strength significantly decreased in the sham group (7.3%, *p*<0.01). Additionally, the percentage of decrease in internal rotation strength was significantly greater in the sham tape group than in the Dynamic tape group, with a large effect size (Table 3). However, between-group differences in flexion and extension percentage strength decline did not reach statistical significance, with small effect sizes observed for external rotation and trivial effect sizes for flexion and extension.

**Table 3:** Percentage of strength decrease

	Dynamic tape		Sham tape		Dominant	Nondominant	Effect size^c^
	Dominant shoulder	Nondominant shoulder	Dominant shoulder	Nondominant shoulder	DT vs ST, *p* value	DT vs ST, *p* value	
Flexion	4.0 ± 17.7	-0.7 ± 13.9	3.7 ± 13.6	-1.4 ± 11.8	0.95	0.65	0.02
Extension	3.7 ± 16.4	-3.0 ± 10.9	1.2 ± 15.6	1.2 ± 14.1	0.66	0.67	0.15
Abduction	4.4 ± 9.0^a^	-2.1 ± 8.4	2.1 ± 9.4	0.9 ± 12.4	0.38	0.35	0.25
Internal rotation	-1.4 ± 10.5	-3.3 ± 12.7	7.3 ± 10.9^a,b^	1.6 ± 10.7	0.03	0.14	0.81
External rotation	2.3 ± 16.6	-2.3 ± 13.5	3.6 ± 14.4	-2.9 ± 14.9	0.79	0.87	0.08

Mean ± standard deviation

*Abbreviations*: *DT* Dynamic tape, *ST* sham tape

^a^For comparisons of different shoulders in the same tape group, *p* < 0.05

^b^For comparisons of different tapes in the same shoulder, *p* < 0.05

^c^Dynamic tape vs sham tape in the dominant shoulder

Pitch velocity and spin rate did not differ significantly between the Dynamic and sham tape groups during sedation. In terms of posttest soreness, while shoulder soreness significantly decreased from posttest day 3 in both the Dynamic tape group (3.4 ± 2.4 on day 1 vs 1.6 ± 1.6 on day 3, *p* < 0.01) and the sham tape group (2.1 ± 1.8 on day 1 vs 1.0 ± 1.3 on day 3, *p* = 0.05), the average posttest visual analog scale (VAS) score did not significantly differ between the two groups at any given time point.

Only 7 participants (35% of the enrolled participants) correctly guessed which session was the Dynamic tape session. The tape force generated by stretching was significantly greater in the Dynamic tape group (0.24 kg vs 0.12 kg, *p* < 0.001).

## Discussion

To the best of our knowledge, this is the first study analyzing the effect of Dynamic tape on shoulder fatigue. Our results demonstrated that in the sham group, the postpitching internal shoulder rotation strength was approximately 93% of the prepitching strength in the resting state, indicating a decrease of 7.3% after 100 pitches.

Dynamic Tape’s ability to stretch in four directions and its viscoelastic properties enable it to conform to the body and assist in complex movements. This support reduces the mechanical load on the muscles and joints, potentially decreasing muscle fatigue. The tape’s strong elastic resistance and recoil act like a bungee cord, decelerating movement and then assisting it, which helps in reducing the overall workload on the muscles [17]. Previous studies have shown that Dynamic Tape significantly reduces hip adduction moment and movement displacement during walking, as well as internal rotation and pelvic obliquity displacement [18]. This suggests that Dynamic Tape provides mechanical benefits by stabilizing the joints and reducing excessive movements that can lead to fatigue and pain.

There are three main proposed mechanisms of action of Dynamic taping: load absorption, force contribution, and movement modification [17]. During the cocking phase of the pitching mechanism, the dominant shoulder gradually rotates externally with a simultaneous increase in the eccentric force provided by the activation of the internal rotators of the shoulder [9, 28]. As Dynamic tape is applied to the maximally internally rotated shoulder, it is put under tension while the shoulder is gradually externally rotated. This could generate tensile support to internal rotators and might be the reason for the decreased fatigue in internal rotation observed in the present study. Alahmari et al. conducted a study evaluating the effect of Dynamic taping on patients with low back pain [20]. Their results also showed that Dynamic tape could control the processes that led to back muscle fatigue. As the effect of Dynamic tape is directional, the taping method used in the present study supported internal rotation only and did not prevent fatigue in other movement directions, such as flexion or abduction.

During the acceleration phase of the pitching mechanism, the internal rotators engage in concentric movement. The energy stored by the Dynamic tape in the cocking phase is released and could contribute to the internal rotation force, as demonstrated by the increased tape-generated internal rotation force observed in the present study. However, despite the potential contribution to internal rotation, no significant differences in pitching speed or spin rate were observed.

The findings that pitch velocity and spin rate remained consistent between the Dynamic and sham tape groups lead us to reconsider the role of individual muscle groups in the context of complex motor tasks. The increased force generated by Dynamic tape, while mitigating muscle fatigue, does not appear to directly enhance the performance metrics of pitch velocity and spin rate. This suggests that the ability to pitch at a consistent speed is influenced by a wide array of factors beyond the support of individual muscle groups.

A possible explanation could be that pitching is a complex movement and that force generation occurs not only from the shoulder but also from the trunk and lower extremities [9]. A study by Yanagisawa and Taniguchi investigated the relationship between lower extremity function and pitching performance and revealed a significant correlation between decreased velocity and decreased hip abduction strength [29]. The implications of these findings suggest that while Dynamic Tape can be beneficial for reducing fatigue and increasing internal rotator strength, additional interventions focusing on improving overall pitching mechanics may be necessary to achieve measurable effects in throwing velocity and spin rate. Future research should consider these factors and explore the combined effects of Dynamic Tape with other performance-enhancing strategies.

In the non-dominant shoulder, we observed a non-significant slight increase in shoulder strength following pitching, a finding consistent with previous research [7]. A potential explanation for this increase could be inadequate warm-up. Although participants were instructed to warm up for 10 min, we did not provide a standardized warm-up protocol, and this duration may have been insufficient. Proper warm-up enhances muscle temperature and blood flow, which can improve muscle strength [30, 31]. In the case of the non-dominant shoulder, the pitching exercise itself might have acted as an extended warm-up, potentially contributing to the observed increase in post-pitching strength.

Delayed onset muscle soreness often follows intense exercise and is thought to stem from microtrauma to muscles and surrounding connective tissue. However, the findings of this study did not indicate a role for Dynamic taping in preventing delayed onset muscle soreness when applied to internal rotation. Although soreness was significantly reduced two days postpitching, no significant differences were observed between the Dynamic and sham taping groups. In contrast, an unpublished study involving 10 subjects by Welch et al. [32] suggested that Dynamic tape could alleviate delayed-onset myalgia in the hamstring complex. However, the motion of the shoulder is more complex and multidirectional than that of the hamstring complex. Muscle soreness during pitching is likely associated with the eccentric activity of shoulder external rotators. As the Dynamic taping method used in this study focused only on supporting internal rotation, other muscles remain susceptible to fatigue, suggesting that internal rotator support alone may not be sufficient to reduce shoulder soreness after pitching.

Shoulder fatigue is closely linked to various shoulder pathologies in pitchers, with internal rotation strength deficits being particularly associated with an increased risk of shoulder injury in overhead athletes [33, 34]. Common pitcher injuries, such as rotator cuff tendinopathy and labral tears, have been linked to excessive external rotation and tensile overload, as well as extreme eccentric load on the bicep–labral complex during the late cocking phase, resulting in external–internal rotation imbalance. The results of our study suggest that taping could be an effective means of reducing internal rotator loading and fatigue, with previous research indicating that reducing fatigue may help prevent shoulder injuries [3, 9, 28, 35, 36]. However, the efficacy of different taping strategies remains a topic of debate. For instance, Kinesio tape primarily acts via neurophysiological mechanisms and may not offer adequate mechanical support [17]. In contrast, Dynamic tape exerts greater mechanical action, with specific taping techniques aimed at supporting particular movement directions. In summary, compared with Kinesio tape, Dynamic tape potentially provides superior mechanical support, load absorption, and movement assistance.

There are several limitations in our study. First, we did not have a control group that pitched without any taping, and the taping method used in the sham group still generated some internal rotation force, as demonstrated by the less pronounced internal rotation fatigue observed in our sham group compared to a previous study by Mullaney et al. [7] This might have resulted in underestimation of the effectiveness of Dynamic taping. Additionally, our study recruited amateur baseball players who played multiple positions rather than professional pitchers. While our results still demonstrated a significant decrease in internal rotation strength, there was considerable variation among participants. Given the differences in pitching mechanics between amateur and professional players, further studies involving more professional pitchers with less variation in strength are necessary to confirm the effect of Dynamic tape [37]. Furthermore, the standardized use of a 4-seam fastball in our study may have limited the generalizability of our findings. Different types of pitches place varying demands on the shoulder and the rest of the kinetic chain [9]. Future studies should consider examining the effects of Dynamic Tape across a variety of pitch types to provide a more comprehensive understanding of its impact on pitching performance. Moreover, it remains unclear whether the internal rotation taping method used in our study is the most effective way to prevent pitching fatigue, as previous research suggests that shoulder fatigue after a baseball game is multidirectional and multifactorial. Finally, we did not exclude patients with intraarticular pathologies such as labral injuries, which may have confounded our results.

## Conclusion

The application of Dynamic tape led to a reduction in shoulder fatigue specific to certain movements, yet it did not lead to significant improvements in pitching performance or the prevention of delayed onset muscle soreness. Although there is potential for Dynamic tape to contribute to the prevention of shoulder injuries, additional research is required to assess the efficacy of various Dynamic taping techniques in alleviating shoulder muscle fatigue.

## Data Availability

The datasets used and/or analyzed during the current study are available from the corresponding author upon reasonable request.

## Data availability

Data is presented within the manuscript and is available upon contacting the corresponding author.
